# Prenatal ultrasound diagnosis and prognosis of persistent left superior vena cava: a 10-year retrospective cohort study at a single center in China

**DOI:** 10.3389/fmed.2026.1743489

**Published:** 2026-02-06

**Authors:** Ronghui Wei, Jingyi Gong, Wen Ling, Qiumei Wu, Guorong Lyu, Yu Wang, Zongjie Weng

**Affiliations:** 1 Department of Medical Ultrasonics, Shishi Maternal and Child Health Hospital, Quanzhou, Fujian, China; 2 Department of Medical Ultrasonics, Fujian Maternity and Child Health Hospital, College of Clinical Medicine for Obstetrics & Gynecology and Pediatrics, Fujian Medical University, Fuzhou, Fujian, China; 3 Department of Medical Ultrasonics, Second Affiliated Hospital of Fujian Medical University, Quanzhou, Fujian, China

**Keywords:** echocardiography, fetus, persistent left superior vena cava, prenatal diagnosis, prognosis

## Abstract

**Objective:**

To describe the prenatal ultrasound characteristics of persistent left superior vena cava (PLSVC) in fetuses and its correlation with related malformations, chromosomal abnormalities, and clinical outcomes.

**Methods:**

A 10-year retrospective analysis of the clinical and ultrasound data of 898 fetuses diagnosed with PLSVC at our center was conducted. Ultrasound characteristics of PLSVC type were summarised systematically, and incidence rates of abnormalities and pregnancy outcomes of PLSVC types were determined.

**Results:**

Diagnosing PLSVC requires the 4CV, 3VV and 3VT views, while auxiliary classification requires parasagittal and innominate vein views. PLSVC ultrasound features include coronary sinus dilation and an additional vascular cross-section on the left side of the pulmonary artery. Types I and II PLSVC involved 94.2% vs. 5.8% of cases, respectively. Type I PLSVC had lower incidence of abnormalities (70.3%) than Type II (100%; *p* < 0.001) and higher birth rates (63.5% vs. 7.7%; *p* < 0.001). However, they differed non-significantly in incidence of chromosomal abnormalities (*p* > 0.05). Of fetuses, 28.0 and 72.0% had isolated and non-isolated PLSVC, respectively. Lower incidence of chromosomal abnormalities occurred in fetuses with isolated PLSVC (7.8%) than that in non-isolated PLSVC (22.0%; *p* < 0.05). Among the non-isolated group, the subgroup with coexisting cardiac and extracardiac abnormalities had the highest incidence of chromosomal abnormalities (39.5%; *p* < 0.005). Higher live birth rate occurred for fetuses with isolated PLSVC (99.2%) than for non-isolated PLSVC (45.1%; *p* < 0.001).

**Conclusion:**

Multifaceted prenatal ultrasound is valuable for classifying and categorizing fetal PLSVC. Classifying PLSVC and assessing accompanying abnormalities is key to determining prognosis. Type II or non-isolated PLSVC, when accompanied by intracardiac and extracardiac abnormalities, requires enhanced genetic testing and multidisciplinary management. Contrariwise, Type I or isolated PLSVC has good prognosis.

## Introduction

Persistent left superior vena cava (PLSVC) is one of the most common abnormalities of the systemic venous system (1, 2). It is estimated to affect approximately 0.3 to 0.5% of the general population (2, 3), but this figure can be as high as 4 to 12% in patients with congenital heart disease (4–6). Previous fetal cardiovascular ultrasound studies have primarily focused on the heart and major arteries, with relatively limited focus on the venous system. However, PLSVC is closely associated with various malformations and adverse pregnancy outcomes, making in-depth research in this area of significant importance (7, 8).

PLSVC has multiple subtypes (9), and current classification primarily relies on the drainage site—Type I (draining into the right atrium) and Type II (draining into the left atrium), each further subdivided into three subtypes based on the presence or absence of the innominate vein and right superior vena cava (10). Existing studies on PLSVC subtypes have primarily focused on embryonic mechanisms (9, 11), with limited large-scale evidence to clearly establish associations between different subtypes and associated abnormalities, chromosomal abnormalities, and prognosis.

PLSVC is associated with both intracardiac and extracardiac abnormalities (3, 12). Berg et al. (12) reported that 82.9% of PLSVC cases were associated with intracardiac abnormalities, while Esin et al. (13) found that 77.5% of PLSVC cases were non-isolated. Multiple studies have reported an association between PLSVC and chromosomal abnormalities (14–16). However, the characteristic differences between different subtypes of PLSVC remain unclear, the association with chromosomal abnormalities has not been systematically validated, and prognostic assessment lacks classification-based guidance.

Therefore, the aim of this study was to describe the ultrasound characteristics and accompanying abnormalities of each type of PLSVC, reveal the correlation between classification and chromosomal abnormalities, and summarize the clinical outcomes of different types of PLSVC.

## Materials and methods

### Study cohort

This retrospective study analyzed the ultrasound and clinical data of 182,124 fetuses who underwent prenatal ultrasound examinations at our hospital between June 2014 and June 2024. Fetuses diagnosed with PLSVC via prenatal ultrasound were included, while cases lacking necessary prenatal or postnatal follow-up data or incomplete ultrasound information were excluded. Diagnoses were confirmed through fetal echocardiography during pregnancy and postnatal verification, resulting in 898 fetuses meeting the study criteria. The detection window period was from 11^+3^ to 39^+6^ weeks, with an average gestational age of (24.3 ± 4.9) weeks. The maternal age range was 16 to 47 years, with an average age of (29.8 ± 5.2) years; all participating mothers signed informed consent forms for prenatal ultrasound examinations. This study was approved by the Ethics Committee of Fujian Provincial Maternal and Child Health Hospital (Approval Number: 2014–043).

### Screening methods and instruments

The high-resolution ultrasound diagnostic equipment used in this study includes the GE Voluson E8, E10, and Philips EPIQ7. The abdominal probe operates at a frequency of 1–6 MHz, the transvaginal probe at 5–9 MHz, and the cardiac probe at 2–8 MHz. All prenatal ultrasound examinations are conducted by physicians qualified in prenatal diagnosis. Abnormal cases are reviewed by two physicians in a double-blind consultation (one of whom must be at least an associate chief physician or above), and a joint diagnostic report is issued.

**Figure 1:**
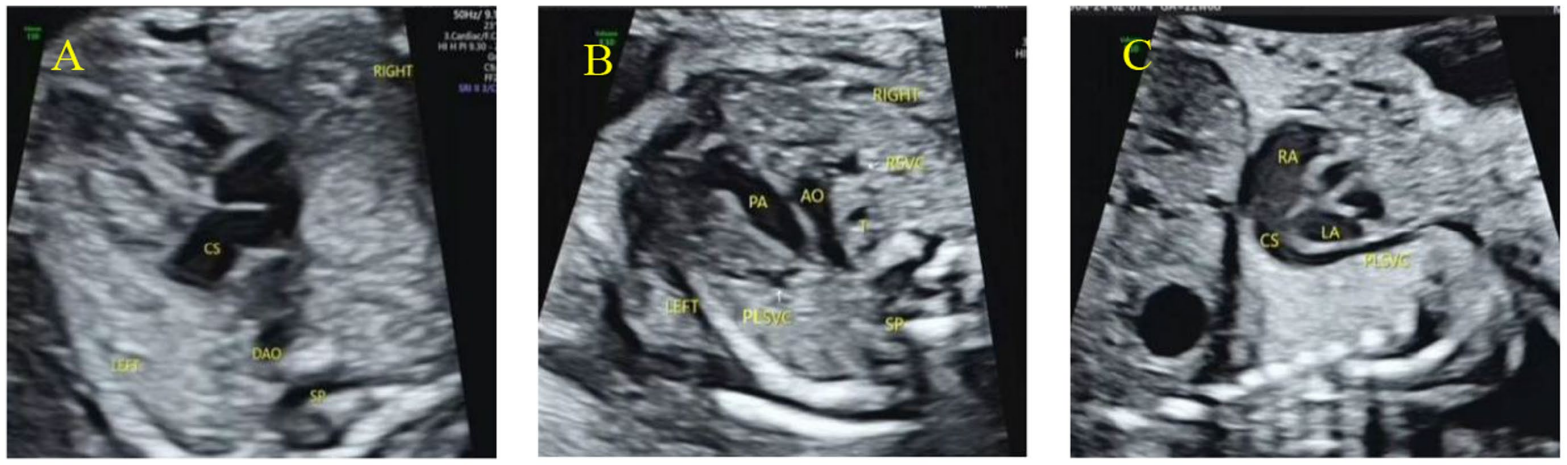
Primary ultrasound sections of fetal PLSVC. **(A)** The coronary sinus section shows the coronary sinus is widened; **(B)** the 3VT section shows the “four-vessel sign,” that is, the left superior vena cava appears on the left side of the pulmonary artery; **(C)** the paravertebral sagittal section of the neck and chest shows the left superior vena cava connecting to the dilated coronary sinus and draining into the right atrium.

Ultrasound examination of fetal PLSVC was performed according to Ultrasound in Obstetrics and Gynecology (ISUOG) recommendations (17, 18), follow the recommendations of the International Society of Ultrasound in Obstetrics and Gynecology (ISUOG) (17, 18): use a six-plane scan in early pregnancy and a nine-plane scan in mid- and late pregnancy. Key planes and findings for diagnosing PLSVC: Four-chamber view: dilation of the coronary sinus (Figure 1A); 3VV or 3VT view: ‘four-vessel sign’ (an additional vascular cross-section on the left side of the pulmonary artery, Figure 1B) or ‘three-vessel sign’ (absence of the right superior vena cava on the right side of the aorta); parasagittal view: demonstrates the course and drainage site of the PLSVC (Figure 1C); innominate vein view: aids in PLSVC classification.

### PLSVC classification and grouping

**Figure 2:**
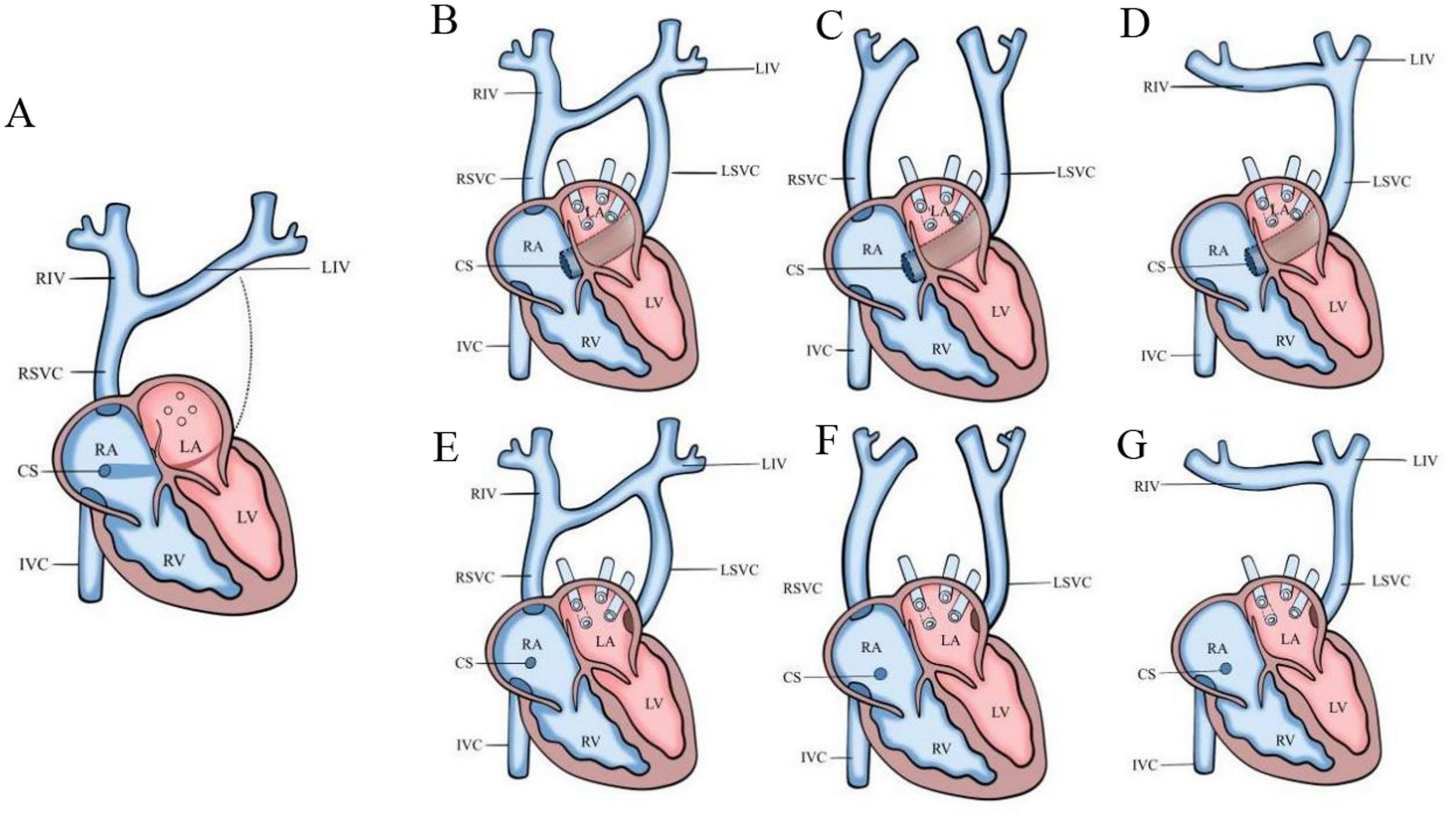
Schematic diagram of normal heart and PLSVC subtyping. **(A)** Normal heart; **(B)** Type Ia PLSVC; **(C)** Type Ib PLSVC; **(D)** Type Ic PLSVC; **(E)** Type IIa PLSVC; **(F)** Type IIb PLSVC; **(G)** Type IIc PLSVC.

Referring to relevant literature (10, 19), based on the LSVC drainage site and the presence or absence of the innominate vein, it is classified as: Type I: drainage through the coronary sinus to the right atrium (Ia/Ib/Ic subtypes); Type II: direct drainage to the left atrium (IIa/IIb/IIc subtypes) (Figure 2). Grouping: Isolated PLSVC group (no concomitant abnormalities); non-isolated PLSVC group (including soft marker abnormalities and/or other cardiac and extracardiac structural anomalies).

### Ultrasound follow-up and management

When prenatal ultrasound reveals a fetus with PLSVC, a multidisciplinary consultation will be initiated to assess prognosis and provide perinatal management recommendations. Pregnant women who choose to continue the pregnancy will undergo genetic testing on an informed consent basis, with test results documented in detail. If the infant is born, a routine physical examination will be conducted, and imaging studies such as echocardiography, CT or MRI will be selected based on the specific circumstances. If pregnancy is terminated, local pathological examination will be performed with the consent of the pregnant woman and her family, and relevant specimens will be properly preserved, documented, and photographed. All fetuses will be validated through postnatal imaging studies, surgery, or pathological examination following pregnancy termination.

### Statistical analysis

Statistical analysis of the collected data was performed using SPSS 22.0 statistical software. If the measurement data followed a normal distribution, it was described using the mean ± standard deviation $\left(\bar{x}\pm s\right)$ . Categorical data were described using frequency and proportion. Intergroup differences were analyzed using the chi-square test or Fisher’s exact probability test. If the overall difference was statistically significant, pairwise comparisons between groups were further conducted using the chi-square split test, and the Bonferroni method was used to set the significance level *α*' to 0.005 (i.e., 0.05/10 = 0.005). For all other analyses, the significance level α was set at 0.05. A *p*-value < 0.05 (two-sided) was considered statistically significant.

## Results

### Classification, grouping, and outcomes of PLSVC

**Figure 3:**
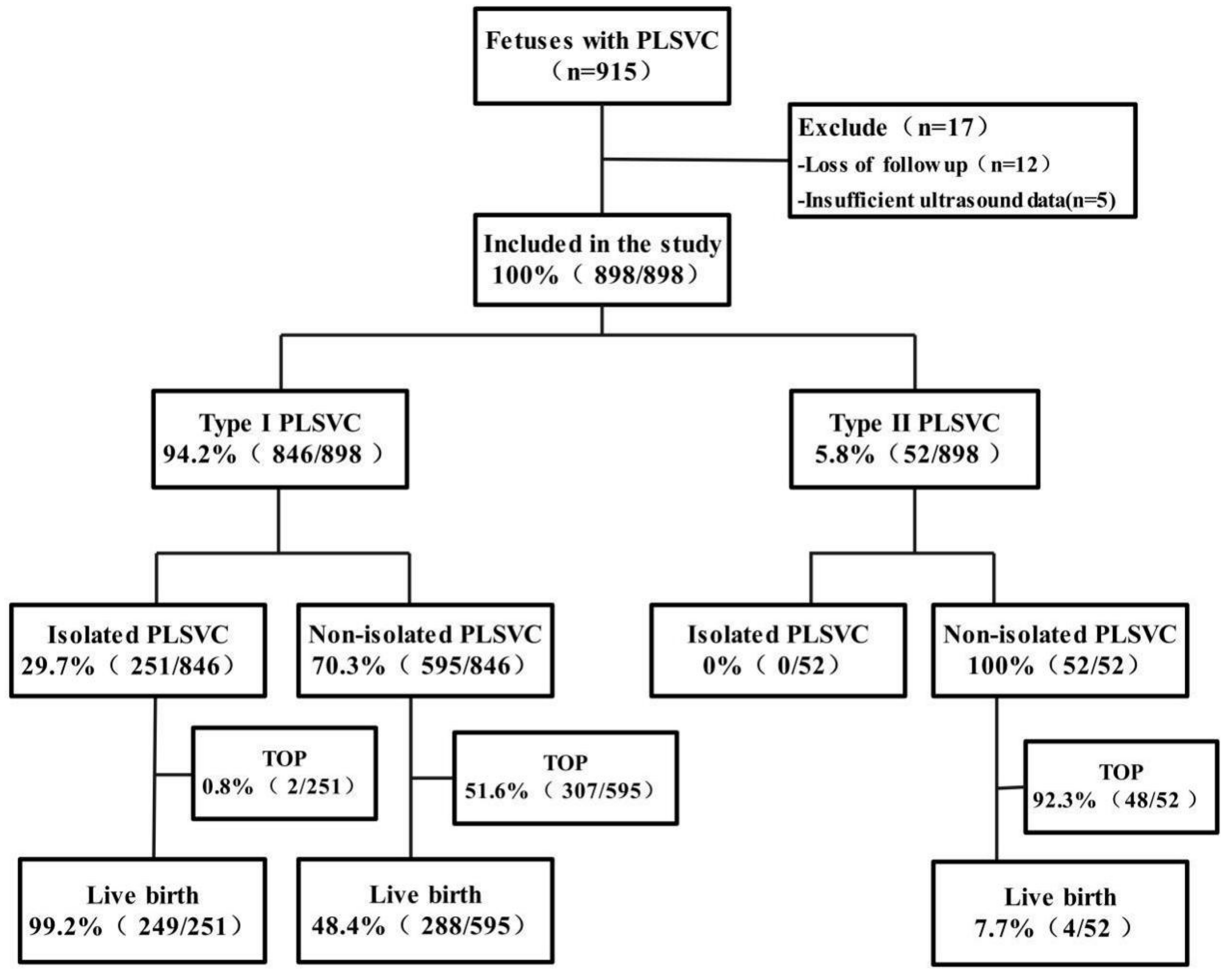
Flow chart of fetuses with PLSVC included in the study.

**Figure 4:**
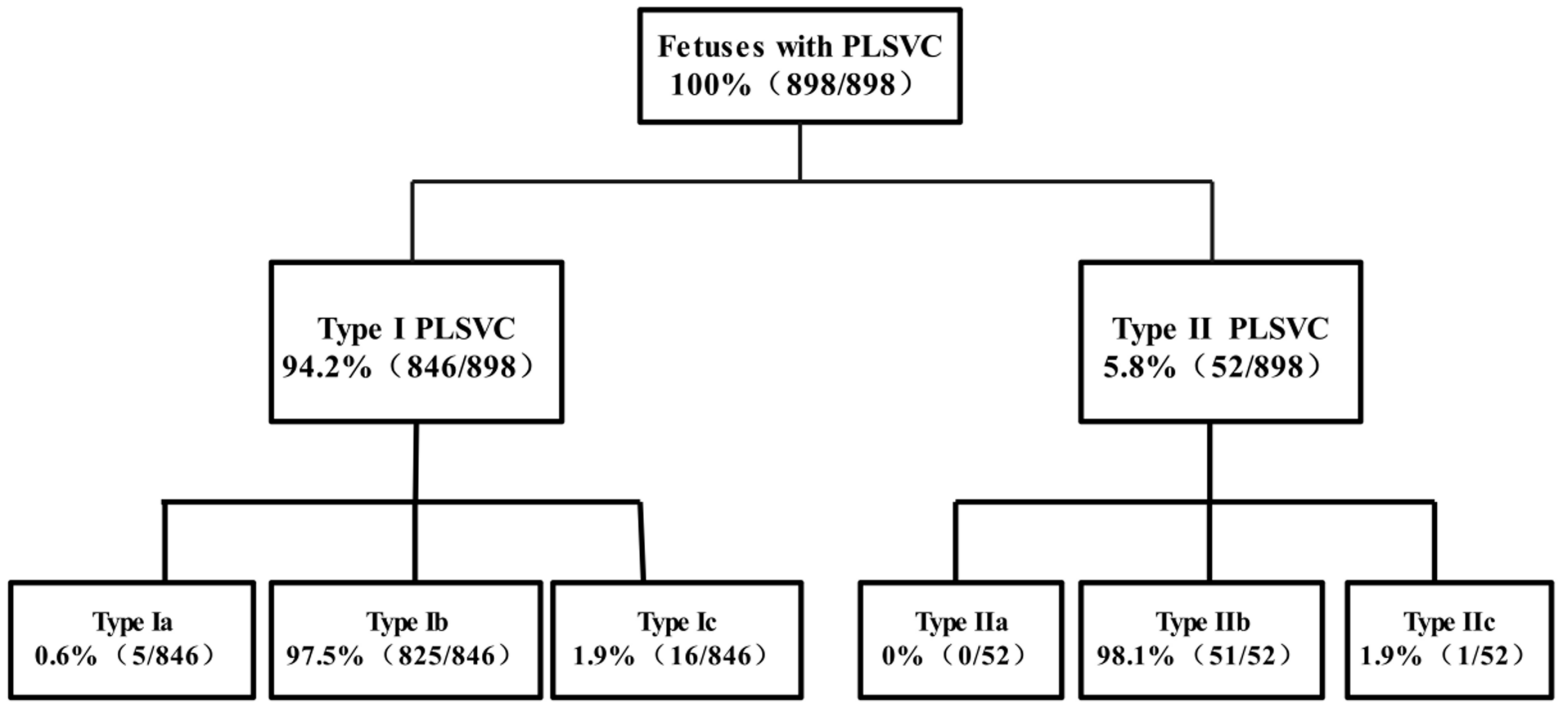
Distribution of foetuses with each subtype of PLSVC.

**Figure 5:**
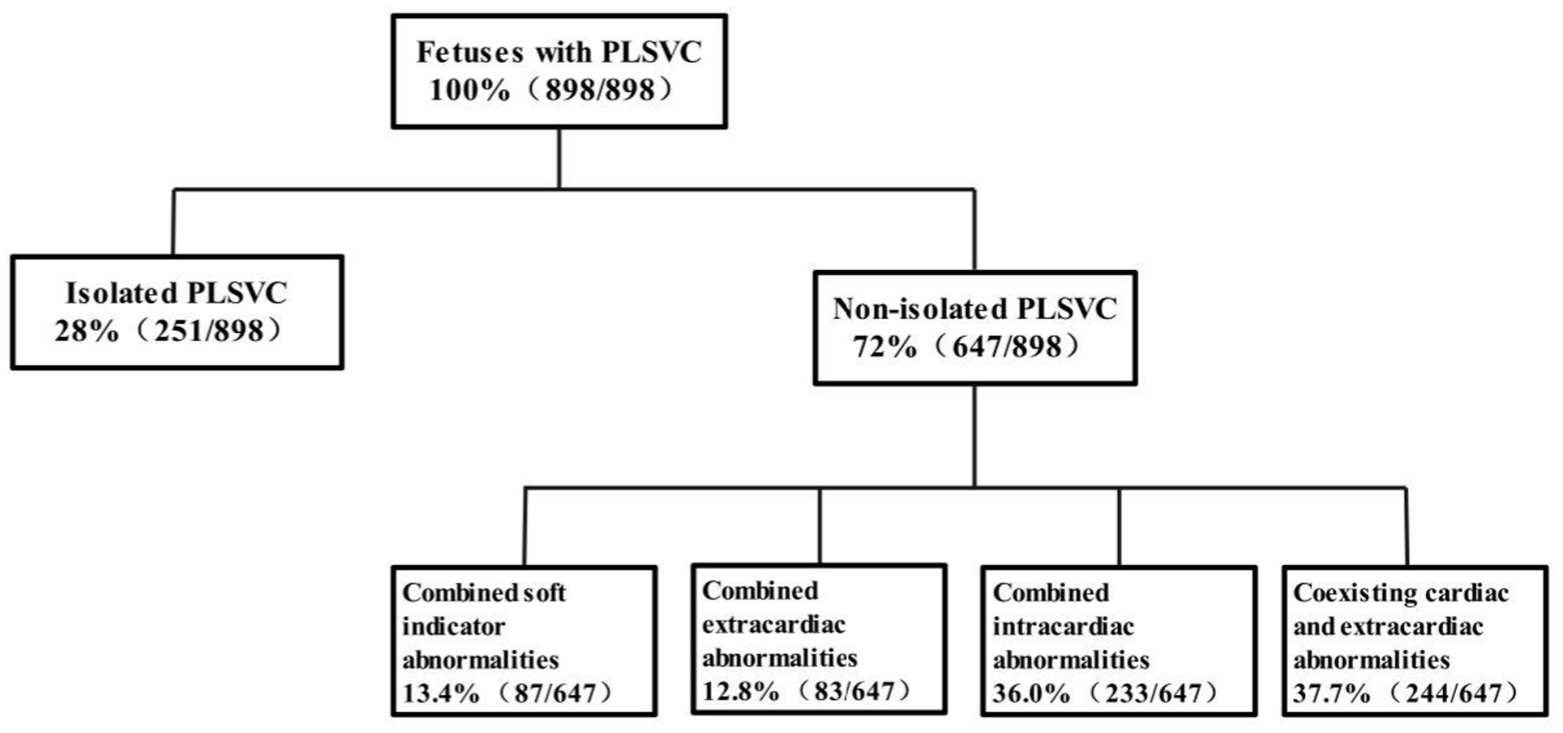
Distribution of PLSVC foetuses in each group.

Among 182,124 fetuses, 898 cases of PLSVC were detected, with a detection rate of 0.5% (898/182,124). 94.2% (846/898) were Type I PLSVC, and 5.8% (52/898) were Type II PLSVC; 28.0% (251/898) were isolated PLSVC, and 72.0% (647/898) were non-isolated PLSVC. Among the 898 confirmed cases, 541 resulted in live births, and 357 resulted in pregnancy termination. A detailed flowchart of case inclusion is provided (Figure 3), and the distribution of fetuses with different PLSVC subtypes and groupings is shown in Figures 4, 5.

### Ultrasound characteristics of each subtype of PLSVC

**Figure 6:**
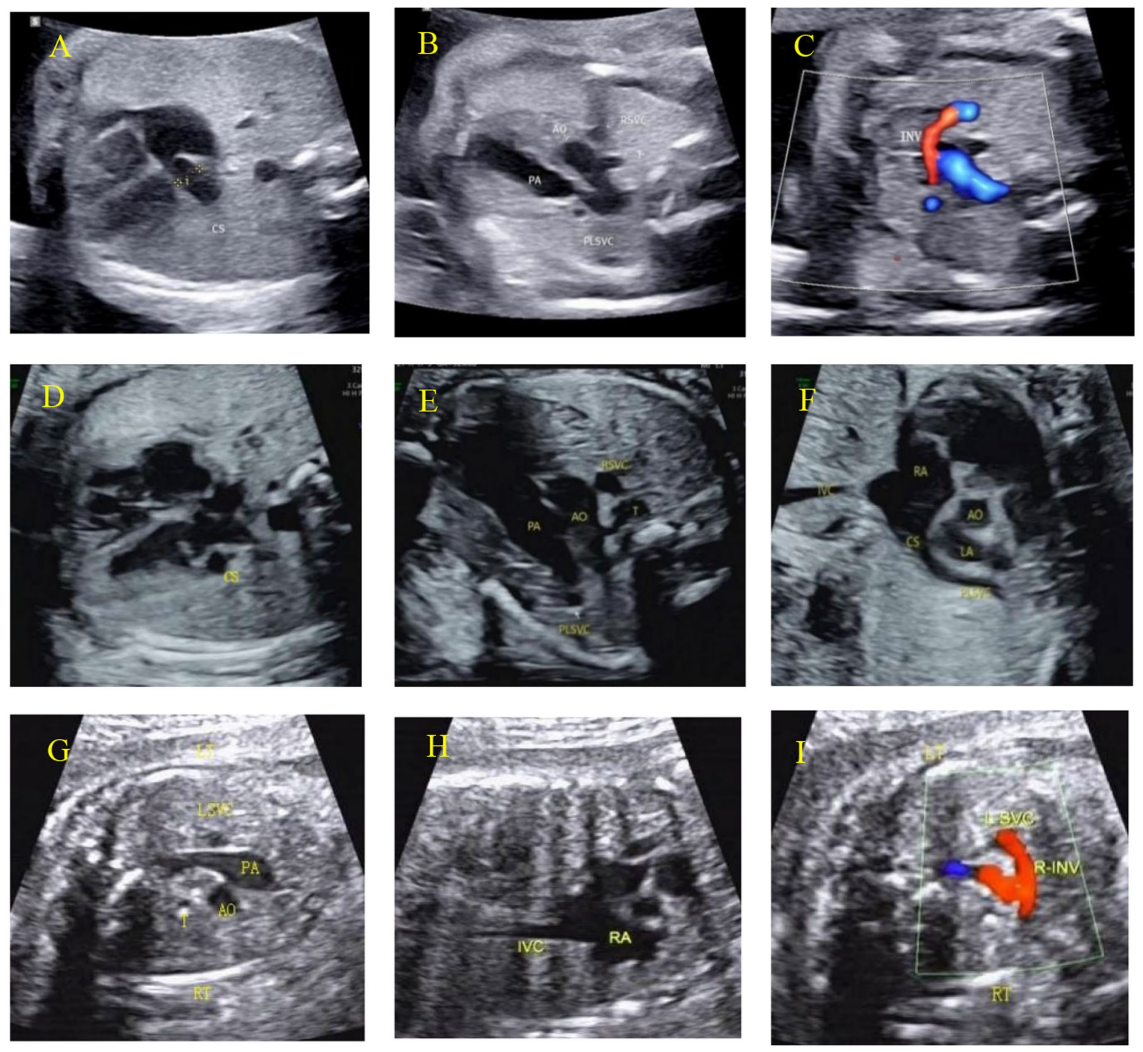
Characteristic ultrasound images of Type I PLSVC: **(A–F)** and **(G–I)**. Type Ia: **(A)** The coronary sinus section shows the coronary sinus is widened; **(B)** the 3VT section shows the “four-vessel sign,” that is, the left superior vena cava appears on the left side of the pulmonary artery; **(C)** shows the innominate vein flowing into the superior vena cava. Type Ib: **(D)** the 4CV section shows the dilated coronary sinus; **(E)** the 3VT section shows the left superior vena cava on the left side of the pulmonary artery; **(F)** the paravertebral sagittal section of the neck and chest shows the left superior vena cava connecting to the dilated coronary sinus and draining into the right atrium. Type Ic: **(G)** the 3VT section shows the left superior vena cava on the left side of the pulmonary artery, and the right superior vena cava is not shown; **(H)** the long-axis section of the superior and inferior vena cava does not show the right superior vena cava; **(I)** CDFI shows the right innominate vein draining into the left superior vena cava on the innominate vein section.

Among 846 cases of Type I PLSVC, 831 cases showed widening of the coronary sinus (Figures 6A,D), while 15 cases did not show widening of the coronary sinus. Type Ia and Type Ib: In the 3VV or 3VT section, they showed the ‘four-vessel sign’ (Figures 6B,E); Type Ic: In the same planes, it presents as the ‘three-vessel sign’ (Figure 6G). In the parasagittal planes: Types Ia and Ib: Both show the left superior vena cava draining into the right atrium via the coronary sinus (Figure 6F); Type Ic: The right superior vena cava is not detected (Figure 6H). In the innominate vein section: Type Ia: the innominate vein is visible (Figure 6C); Type Ib: the innominate vein is absent; Type Ic: the right innominate vein is visible (Figure 6I).

**Figure 7:**
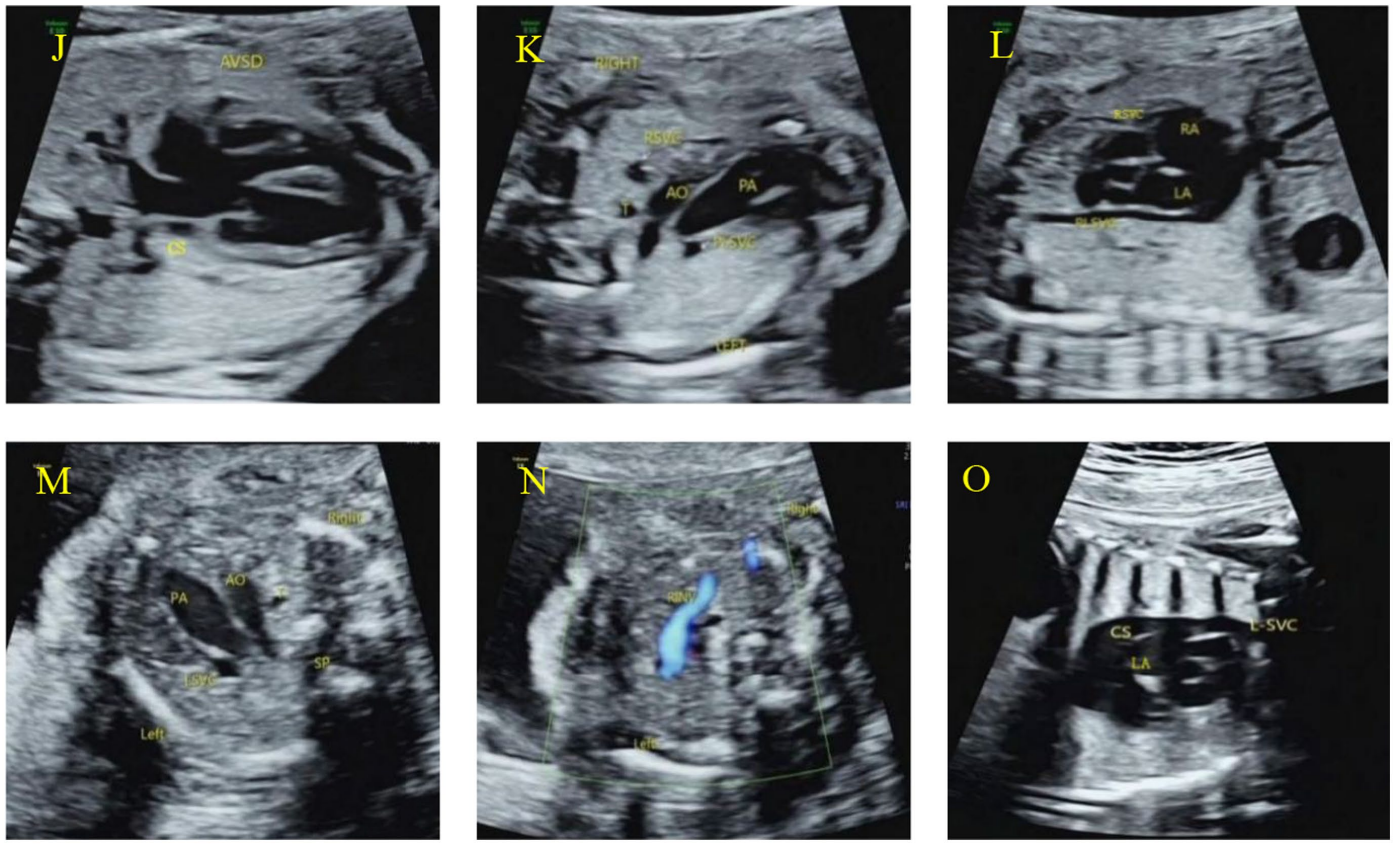
Characteristic ultrasound images of Type II PLSVC: **(J–L)** and **(M-O)**. Type IIb: **(J)** The 4CV section shows atrioventricular septal defect and coronary sinus; **(K)** the 3VT section shows the left superior vena cava on the left side of the pulmonary artery; **(L)** the paravertebral sagittal section of the neck and chest shows the left superior vena cava draining into the left atrium. Type IIc: **(M)** The 3VT section shows the left superior vena cava on the left side of the pulmonary artery, and the right superior vena cava is not shown; **(N)** CDFI shows the right innominate vein draining into the left superior vena cava on the innominate vein section; **(O)** the paravertebral sagittal section of the neck and chest shows the left superior vena cava draining into the left atrium.

All 52 cases of Type II PLSVC showed no significant widening of the coronary sinus. Among these, 51 cases of Type IIb exhibited the ‘four-vessel sign’ in the 3VV or 3VT planes (Figure 7K); 1 case of Type IIc exhibited the ‘three-vessel sign’ (Figure 7M); in the parasagittal section, all cases showed the left superior vena cava draining into the left atrium (Figures 7L,O); in the innominate vein section, only 1 case showed the right innominate vein (Figure 7N).

### Combined intracardiac and extracardiac abnormalities

Classified by subtype: The proportion of Type I PLSVC cases with abnormalities was 70.3% (595/846); 100% of Type II PLSVC cases (52/52) had abnormalities, with 88.5% (46/52) having heterotaxy syndrome. The incidence of abnormalities in Type I PLSVC (70.3%) was significantly lower than that in Type II (100%; *p* < 0.001; Table 1).

**Table 1:** Comparison of associated abnormalities, live birth rates, and chromosomal abnormality rates in fetuses with Types I and II PLSVC [*n* (%)].

Project typing	Type I PLSVC	Type II PLSVC	*χ* ^2^	*p*
Associated abnormalities	595 (70.3)	52 (100)	21.41	<0.001
Live births	537 (63.5)	4 (7.7)	63.65	<0.001
Chromosomal abnormalities	56 (19.1)	3 (17.6)	0.02	0.880^#^

# Chi-square test with continuity correction was applied.

Classification by group: Among 898 PLSVC fetuses, 28.0% (251/898) were classified as isolated cases, and 72.0% (647/898) belonged to the non-isolated group; among the non-isolated group, the subgroup with coexisting cardiac and extracardiac abnormalities had the highest proportion at 37.7% (244/647). Common cardiac abnormalities included ventricular septal defect, aortic arch stenosis, and heterotaxy syndrome; common soft marker abnormalities included single umbilical artery; common extracardiac abnormalities included gastrointestinal system abnormalities and central nervous system abnormalities.

### Combined chromosomal abnormalities

The overall incidence of chromosomal abnormalities in fetuses with PLSVC was 19.0% (59/310). The most common types of chromosomal abnormalities included trisomy 21, Turner syndrome, and trisomy 18. Among 64 cases of isolated PLSVC fetuses, 5 cases were found to have chromosomal abnormalities, with an incidence rate of 7.8%. The incidence of non-isolated PLSVC chromosomal abnormalities was 22.0% (54/246). The incidence rate of chromosomal abnormalities in the isolated group (7.8%) was lower than that in the non-isolated group (22.0%; *p* < 0.05; Table 2). In the non-isolated group, the incidence of chromosomal abnormalities was highest (39.5%) in the subgroup with concurrent intracardiac and extracardiac abnormalities (*p* < 0.005; Table 2). The incidence of fetal chromosome abnormalities was 19.1% (56/293) for Type I PLSVC and 17.6% (3/17) for Type II PLSVC. There was no significant difference in the incidence of chromosomal abnormalities between Type I (19.1%) and Type II (17.6%; *p* > 0.05; Table 1).

**Table 2:** Comparison of the incidence of chromosomal abnormalities in PLSVC fetuses in each group.

Groups	Total number of cases	Number of chromosomal abnormalities	Occurrence (rate, %)	*χ* ^2^	*p*
Without associated anomalies	64	5	7.8	30.307	<0.05
Combined Extracardiac anomaly	41	3	7.3		
Combined ultrasound soft marker abnormality	36	5	13.9		
Combined intracardiac anomaly	93	16	17.2		
Also combined intra- and extracardiac abnormalities	76	30	39.5^a^^b^		

a Comparison with the PLSVC group without associated anomalies, *p* < 0.005.

b Comparison with the group with extracardiac anomalies, *p* < 0.005.

No significant differences were observed among the other groups in pairwise comparisons.

### Outcome and prognosis

Among 898 fetuses with PLSVC, 60.2% (541/898) were live births, and 39.8% (357/898) resulted in pregnancy termination. The live birth rate for Type I PLSVC (63.5%) was significantly higher than that for Type II (7.7%; *p* < 0.001; Table 1). The live birth rate in the isolated group (99.2%) was higher than that in the non-isolated group (45.1%; *p* < 0.001; Table 3); For specific conditions of live births in non-isolated groups, please refer to Appendix 1; among the non-isolated group, the subgroup with concurrent intracardiac and extracardiac abnormalities had the lowest live birth rate (20.9%; *p* < 0.005; Table 3).

**Table 3:** Comparison of live birth rates among groups.

Groups	Total number of cases	Live births	Live birth (rate, %)	*χ* ^2^	*p*
Without associated anomalies	251	249	99.2	361.239	<0.05
Combined ultrasound soft marker abnormality	87	76	87.4^a^		
Combined extracardiac anomaly	83	55	66.3^a^^b^		
Combined intracardiac anomaly	233	110	47.2^a^^b^^c^		
Also combined intra- and extracardiac abnormalities	244	51	20.9^a^^b^^c^^d^		

a A significant difference compared to the isolated PLSVC group (*p* < 0.005).

b A significant difference compared to the group with ultrasound soft marker abnormalities (*p* < 0.005).

c A significant difference compared to the group with extracardiac anomalies (*p* < 0.005).

d A significant difference compared to the group with intracardiac anomalies (*p* < 0.005).

## Discussion

The formation of PLSVC originates from the failure of the left anterior vena cava to regress during embryonic development (approximately weeks 7–8) (19). In this 10-year retrospective study, 898 fetuses with a prenatal diagnosis of persistent left superior vena cava (PLSVC) were analyzed to evaluate sonographic features, anatomical subtypes, associated anomalies, and perinatal outcomes. Most cases were classified as Type I PLSVC (94.2%), whereas Type II PLSVC accounted for a smaller proportion (5.8%). Type II PLSVC was more frequently associated with structural abnormalities and showed an extremely low live birth rate. Clinical outcomes differed according to PLSVC subtype and the presence of concomitant anomalies. Accurate prenatal diagnosis of PLSVC is therefore important for genetic counseling and clinical decision-making.

The 4CV, 3VV, and 3VT views are commonly used ultrasound screening views for PLSVC (19, 20), while the parasagittal view and innominate vein view are of significant value for PLSVC classification and differential diagnosis. Our study found that the dilation of the coronary sinus observed on the 4CV is a key diagnostic feature of PLSVC. The ‘four-vessel’ sign observed on the 3VV or 3VT planes is not an exclusive characteristic of PLSVC; supraventricular pulmonary venous malformation can also present with this sign (PLSVC blood flow is centripetal, while supraventricular pulmonary venous malformation blood flow is centrifugal). Additionally, when accompanied by absence of the right superior vena cava, only three vessels are visible (21). In summary, ultrasound diagnosis of PLSVC requires multi-plane combined assessment.

PLSVC has multiple subtypes. Azizova et al. (9) classified PLSVC into Type IV. We found that observing the communication between the fetal coronary sinus and the atrium, as well as the connection between the left superior vena cava and the left pulmonary vein, is challenging in routine ultrasound examinations. Therefore, this study classified PLSVC into Types I and II based on the drainage site of the LSVC (10), aiming to simplify the diagnosis of PLSVC during the fetal period.

The detection rate of PLSVC in this study was 0.5%, which is consistent with previous literature reports (22). Type I PLSVC was more common (94.2%), while Type II was rare (5.8%), similar to the findings of Li et al. (23). Types Ic and IIc (absence of the right superior vena cava) are rare subtypes, with only 17 cases detected (0.009%), lower than the results reported by Lopes et al. (24, 25), which may be related to racial differences. There are few reports on the association between different subtypes of PLSVC and fetal structural abnormalities (26). We found that 70.3% of Type I PLSVC cases were associated with abnormalities, while all Type II cases were associated with abnormalities, particularly heterotaxy syndrome (88.5%), similar to the findings of Berg et al. (12), suggesting a possible association between this subtype and fetal lateralization abnormalities. Additionally, the live birth rate of Type I PLSVC fetuses was significantly higher than that of Type II PLSVC, indicating that PLSVC classification is valuable for risk of TOP. Notably, a significant proportion (48.4%) of non-isolated Type I PLSVC cases opted for continued pregnancy. This study categorized cases with abnormal soft markers as non-isolated, as these fetuses are often assessed as having relatively favorable prognoses during prenatal genetic counseling and follow-up, leading families to prefer continuing the pregnancy. Additionally, among Type I PLSVC fetuses with concurrent cardiac structural anomalies and ultimately live births, a higher proportion were associated with ventricular septal defects or aortic arch constriction/stenosis. These two types of cardiac anomalies are generally considered to have better interventional feasibility and long-term prognosis during prenatal evaluation, resulting in relatively lower pregnancy termination rates. The specific details of all live-born non-isolated PLSVC fetuses are summarized in a table in Appendix 1.

Among the abnormalities associated with PLSVC, the most common soft marker abnormality was single umbilical artery, common cardiac abnormalities included ventricular septal defect, aortic arch narrowing, and heterotaxy syndrome, and common extracardiac abnormalities included digestive system abnormalities and central nervous system abnormalities, consistent with previous studies (5, 12, 14). Therefore, when PLSVC is detected, a comprehensive structural assessment of the fetus is necessary to rule out other potential abnormalities.

Studies have shown an association between PLSVC and chromosomal abnormalities (14–16, 27). In this study, the rate of chromosomal abnormalities in fetuses with isolated PLSVC was 7.8%, which is similar to the findings of Gustapane et al. (3) (7.0%), primarily manifested as microdeletions/microduplications, with an incidence rate higher than that in the general population with normal ultrasound findings [approximately 1.0–1.5% (28)]. Therefore, whether chromosomal testing is necessary when isolated PLSVC is detected warrants further discussion. In fetuses with non-isolated PLSVC, the highest risk of chromosomal abnormalities is observed when combined with intracardiac and extracardiac anomalies, which may be related to the fact that genetic syndromes often coexist with both intracardiac and extracardiac abnormalities. Hu et al. (29) reported that the risk of chromosomal abnormalities increases with the number of ultrasound soft markers present in the fetus. In this study, the rate of chromosomal abnormalities in cases of PLSVC combined with abnormal ultrasound soft markers was 13.9%, and all cases of chromosomal abnormalities in this group were accompanied by multiple abnormal ultrasound soft markers. Therefore, genetic testing should be recommended for cases of PLSVC combined with multiple abnormal ultrasound soft markers.

## Limitation

Among the limitations of the study is its retrospective design, spanning a prolonged time; therefore, some early data may contain certain errors. Not all fetuses underwent genetic testing, and when major malformations were present, pregnant women were more likely to terminate the pregnancy directly, leading to bias in the study data. Additionally, some subtypes had small sample sizes (e.g., only one case of Type IIc), necessitating multi-center validation.

## Conclusion

Multisection prenatal ultrasound is of great value in the classification and categorization of fetal PLSVC. The classification of PLSVC and the assessment of accompanying abnormalities are key to determining prognosis. Type II or non-isolated PLSVC (especially when accompanied by intracardiac and extracardiac abnormalities) requires enhanced genetic testing and multidisciplinary management, while Type I or isolated PLSVC has a good prognosis.

## Data Availability

The raw data supporting the conclusions of this article will be made available by the authors, without undue reservation.

## References

[1] Povoski SP Khabiri H. Persistent left superior vena cava: review of the literature, clinical implications, and relevance of alterations in thoracic central venous anatomy as pertaining to the general principles of central venous access device placement and venography in cancer patients. World J Surg Oncol. (2011) 9:173. doi: 10.1186/1477-7819-9-173 22204758 PMC3266648

[2] Sheikh AS Mazhar S. Persistent left superior vena cava with absent right superior vena cava: review of the literature and clinical implications. Echocardiography. (2014) 31:674–9. doi: 10.1111/echo.12514 24460570

[3] Gustapane S Leombroni M Khalil A Giacci F Marrone L Bascietto F . Systematic review and meta-analysis of persistent left superior vena cava on prenatal ultrasound: associated anomalies, diagnostic accuracy and postnatal outcome. Ultrasound Obstet Gynecol. (2016) 48:701–8. doi: 10.1002/uog.15914, 26970258

[4] Yagel S Kivilevitch Z Cohen SM Valsky DV Messing B Shen O . The fetal venous system, part I: normal embryology, anatomy, hemodynamics, ultrasound evaluation and Doppler investigation. Ultrasound Obstet Gynecol. (2010) 35:741–50. doi: 10.1002/uog.7618 20205155

[5] Galindo A Gutiérrez-Larraya F Escribano D Arbues J Velasco JM. Clinical significance of persistent left superior vena cava diagnosed in fetal life. Ultrasound Obstet Gynecol. (2007) 30:152–61. doi: 10.1002/uog.4045 17616965

[6] Poenaru MO Hamoud BH Sima RM Valcea ID Chicea R Ples L. Persistent left superior vena cava significance in prenatal diagnosis-case series. J Clin Med. (2022) 11:4020. doi: 10.3390/jcm11144020 35887792 PMC9316240

[7] Achiron R Hegesh J Yagel S Lipitz S Cohen SB Rotstein Z. Abnormalities of the fetal central veins and umbilico-portal system: prenatal ultrasonographic diagnosis and proposed classification. Ultrasound Obstet Gynecol. (2000) 16:539–48. doi: 10.1046/j.1469-0705.2000.00220.x 11169348

[8] Durand I Hazelzet T Gillibert A Parrod C David N El Youssef F . Outcomes following prenatal diagnosis of isolated persistent left superior vena cava. Arch Cardiovasc Dis. (2022) 115:335–47. doi: 10.1016/j.acvd.2022.03.005, 35660361

[9] Azizova A Onder O Arslan S Ardali S Hazirolan T. Persistent left superior vena cava: clinical importance and differential diagnoses. Insights Imaging. (2020) 11:110. doi: 10.1186/s13244-020-00906-2 33057803 PMC7561662

[10] Shengli L Guoyang L. Prenatal ultrasound diagnosis of fetal malformations. 2nd ed. Beijing: Science Press (2017).

[11] Sinkovskaya E Abuhamad A Horton S Chaoui R Karl K. Fetal left brachiocephalic vein in normal and abnormal conditions. Ultrasound Obstet Gynecol. (2012) 40:542–8. doi: 10.1002/uog.11166 22461379

[12] Berg C Knüppel M Geipel A Kohl T Krapp M Knöpfle G . Prenatal diagnosis of persistent left superior vena cava and its associated congenital anomalies. Ultrasound Obstet Gynecol. (2006) 27:274–80. doi: 10.1002/uog.2704 16456841

[13] Esin D Aslan Çetin B Şenol G Selçuki NFT Gedik Özköse Z Acar Z . Clinical significance of prenatally diagnosed persistent left superior vena cava. J Gynecol Obstet Hum Reprod. (2022) 51:102332. doi: 10.1016/j.jogoh.2022.102332, 35123124

[14] Özsürmeli M Büyükkurt S Sucu M Arslan E Akçabay Ç Mısırlıoğlu S . Prenatal diagnosis of persistent left superior vena cava: a retrospective study of associated congenital anomalies. Turk J Obstet Gynecol. (2019) 16:23–8. doi: 10.4274/tjod.galenos.2019.02679, 31019836 PMC6463432

[15] Yang X Su XH Zeng Z Fan Y Wu Y Guo LL . Integrated analysis of comorbidity, pregnant outcomes, and amniotic fluid cytogenetics of fetuses with persistent left superior vena cava. World J Cardiol. (2023) 15:500–7. doi: 10.4330/wjc.v15.i10.500, 37900905 PMC10600788

[16] Du L Xie HN Zhu YX Li LJ Peng R Zheng J. Fetal persistent left superior vena cava in cases with and without chromosomal anomalies. Prenat Diagn. (2014) 34:797–802. doi: 10.1002/pd.4380 24711103

[17] Salomon LJ Alfirevic Z Bilardo CM Chalouhi GE Ghi T Kagan KO . ISUOG practice guidelines: performance of first-trimester fetal ultrasound scan. Ultrasound Obstet Gynecol. (2013) 41:102–13. doi: 10.1002/uog.12342.18, 23280739

[18] Carvalho JS Allan LD Chaoui R Copel JA DeVore GR Hecher K . ISUOG practice guidelines (updated): sonographic screening examination of the fetal heart. Ultrasound Obstet Gynecol. (2013) 41:348–59. doi: 10.1002/uog.12403, 23460196

[19] Chaoui R Heling KS Karl K. Ultrasound of the fetal veins part 2: veins at the cardiac level. Ultraschall Med. (2014) 35:302–18. doi: 10.1055/s-0034-1366848 25127225

[20] Chaoui R Heling KS Kalache KD. Caliber of the coronary sinus in fetuses with cardiac defects with and without left persistent superior vena cava and in growth-restricted fetuses with heart-sparing effect. Prenat Diagn. (2003) 23:552–7. doi: 10.1002/pd.626, 12868081

[21] Raisi-Estabragh Z Khanji MY. A rare case of persistent left superior vena cava with absent right superior vena cava. Eur Heart J. (2020) 41:194–4. doi: 10.1093/eurheartj/ehz466, 31257401

[22] Choi EY Hong SK Jeong NY. Clinical characteristics of prenatally diagnosed persistent left superior vena cava in low-risk pregnancies. Prenat Diagn. (2016) 36:444–8. doi: 10.1002/pd.4801, 26934675 PMC5071676

[23] Li TG Wu WR Ma B Yan ZH Niu KX. Prenatal diagnosis of persistent left superior vena cava using high-definition flow render mode and spatiotemporal image correlation. J Ultrasound Med. (2024) 43:2177–85. doi: 10.1002/jum.16550 39136224

[24] Lopes KRM Bartsota M Doughty V Carvalho JS. Single left superior vena cava: antenatal diagnosis, associated anomalies and outcomes. Ultrasound Obstet Gynecol. (2022) 60:640–5. doi: 10.1002/uog.24966, 35656845 PMC9828089

[25] Sayici UI Ari ME. Persistent left superior vena cava without right superior vena cava during fetal life. Cardiol Young. (2023) 33:2122–3. doi: 10.1017/S1047951123001014, 37127650

[26] Kahramanoglu O Demirci O Uygur L Erol N Schiattarella A Rapisarda AMC. Persistent left superior vena cava with and without right superior vena cava: significance of prenatal diagnosis. Pediatr Cardiol. (2024) 45:377–84. doi: 10.1007/s00246-023-03353-0, 38103069

[27] Cao Q Zhen L Pan M Han J Yang X Xu LL . Prenatal persistent left superior vena cava in low population: not a benign vascular anomaly. Taiwan J Obstet Gynecol. (2022) 61:459–63. doi: 10.1016/j.tjog.2022.03.011, 35595438

[28] Stern S Hacohen N Meiner V Yagel S Zenvirt S Shkedi‐Rafid S . Universal chromosomal microarray analysis reveals high proportion of copy-number variants in low-risk pregnancies. Ultrasound Obstet Gynecol. (2021) 57:813–20. doi: 10.1002/uog.22026, 32202684

[29] Hu T Tian T Zhang Z Wang J Hu R Xiao L . Prenatal chromosomal microarray analysis in 2466 fetuses with ultrasonographic soft markers: a prospective cohort study. Am J Obstet Gynecol. (2020) 224:516.e1–516.e16. doi: 10.1016/j.ajog.2020.10.039, 33122027

