# Supplementary material for: Prenatal ultrasound diagnosis and prognosis of persistent left superior vena cava: a 10-year retrospective cohort study at a single center in China
Source: Front Med (Lausanne). 2026 Feb 6;13:1743489. doi: 10.3389/fmed.2026.1743489 (PMC12920434; doi:10.3389/fmed.2026.1743489)
Supplement: Supplementary file 1 [file Data_Sheet_1.PDF]

Appendix 1:

Table Live Births with Non-isolated PLSVC

Non-isolated PLSVC	Malformations / Soft Markers	Number of live births of type I	Number of live births of type II	Total live births
Combined with ultrasound soft index abnormality	Single umbilical artery	21		76
	Intracardiac echogenic focus	15		
	Pyelectasis	12		
	Mild tricuspid regurgitation	10		
	Hypoplasia of nasal bone	9		
	Local intestinal echo enhancement	4		
	Mild lateral ventricular widening	2		
	Accessory renal artery	2		
	Omphalocele	1		
Combined with intracardiac anomaly	Ventricular septal defect	50	1	110
	Coarctation of the aorta /narrow	29		
	Aberrant Right Subclavian Artery	6		
	Right aortic arch with left subclavian artery deviation	6		
	Tetralogy of Fallot	5		
	Double outlet of right ventricle	4		
	Right aortic arch with mirror-image branches	2		
	Slightly narrowed pulmonary artery	2		
	Tortuosity of the descending aorta	2		
	Dextrocardia	1		
	Hemi-accessory azygos, pericardial effusion	1		
	Left heart hypoplasia	1		
Combined with extracardiac anomaly	permanent right umbilical vein	9		55
	Abnormal size of the gallbladder or gastric bubble (too small, too large, or not visible)	7		
	Ventriculomegaly	6		
	Dilated the intra-abdominal segment of the umbilical vein	4		
	Hydronephrosis	4		
	Talipes equinovarus	2		
	Unilateral renal hypoplasia	2		
	Ectopic kidney	2		
	Small cerebellar vermis	2		
	Low-lying conus medullaris	2		
	Hemivertebrae	2		
	Cleft lip	2		
	Abdominal cyst	1		
	Hepatosplenomegaly with ascites	1		
	Congenital cyst of common bile duct	1		

	Horseshoe kidney	1	
	Bilateral subependymal cysts	1	
	Meconium peritonitis	1	
	Abnormal morphology of the external genitalia	1	
	Lymphatic cyst of occiput	1	
	Duodenal ileus	1	
	Postaxial polydactyly	1	
	Pulmonary cystadenoma	1	
Combined with intra-and extracardiac abnormalities	Ventricular septal defect and abnormal kidney Size/hydronephrosis/ectopic kidney	7	51
	Ventricular septal defect and abnormal size of gallbladder or gastric bubble	3	
	Ventricular septal defect and cleft lip	2	
	Ventricular septal defect and enlarged posterior fossa	2	
	Ventricular septal defect and dilated lateral ventricles	2	
	Ventricular septal defect and visceral inversion	2	
	Ventricular septal defect and low-lying conus medullaris	1	
	Ventricular septal defect and unilateral pulmonary cystadenoma	1	
	Ventricular septal defect and small cerebellar vermis	1	
	Ventricular septal defect and abnormal drainage of ductus venosus	1	
	Ventricular septal defect and bilateral microtia	1	
	Coarctation of the aorta /narrow and abnormal kidney size/hydronephrosis/ectopic kidney	5	
	Coarctation of the aorta and enlarged posterior cranial fossa pool	3	
	Narrowed aortic arch and absent gallbladder	2	
	Aortic arch stenosis and thymic hypoplasia	1	
	Coarctation of the aorta and dacryocystitis	1	
	Narrowing of the aortic arch and enlarged lateral ventricles	1	
	Narrow aortic arch and intrahepatic portal-venous shunt	1	
	Coarctation of the aorta and low-lying conus medullaris	1	

Left atrial isomerism		2
Right atrial isomerism		1
Absence of the hepatic segment of the inferior vena cava	1	
Aberrant Right Subclavian Artery and hemivertebra	1	
Right-sided aortic arch with left subclavian artery deviation and ventriculomegaly	2	
Tetralogy of Fallot and ventriculomegaly	2	
Tetralogy of Fallot and micrognathia	1	
Tetralogy of Fallot and small cerebellar vermis	1	
Tetralogy of Fallot and horseshoe kidney	1	
Tetralogy of Fallot and absence of the right kidney	1	
